# Phosphorylation at S1288 of leukemia associated RhoGEF (LARG/ARHGEF12) induces plasma membrane localization and promotes binding and activation of RhoA

**DOI:** 10.1016/j.jbc.2025.110996

**Published:** 2025-12-01

**Authors:** Won Seok Yang, Neda Z. Ghanem, Steven D. Scahill, Maisel J. Caliva, Hannah Röttig, Annika Fitz, Michelle L. Matter, Joe W. Ramos

**Affiliations:** 1 Department of Interdisciplinary Oncology, LSUHSC School of Medicine, New Orleans, Louisiana, USA; 2 Cancer Biology Program, University of Hawaii Cancer Center, University of Hawaii at Mānoa, Honolulu, Hawaii, USA; 3 Biological Chemistry Program, Department of Biotechnology, Mannheim University of Applied Sciences, Mannheim, Germany; 4 Tulane Cancer Center, School of Medicine, Tulane University, New Orleans, Louisiana, USA

**Keywords:** ARHGEF12, RhoA, LARG, cell invasion, signal transduction, RSK2

## Abstract

Leukemia-associated RhoGEF (LARG) is a guanine nucleotide exchange factor (GEF) known for its specificity toward Ras homolog family member A (RhoA). LARG plays a crucial regulatory role in various cellular processes such as migration, proliferation, invasion, and metastasis by facilitating the exchange of GDP to GTP on RhoA. Phosphorylation of LARG at S1288 by ribosomal S6 kinase 2 (RSK2) promotes RhoA activation. However, the precise mechanism remains unclear. Here, we demonstrate the essential role and mechanism by which S1288 phosphorylation facilitates LARG-mediated invasiveness in response to epidermal growth factor (EGF). Upon EGF stimulation, RSK2 phosphorylates LARG at S1288, thereby promoting the membrane translocation of LARG. Furthermore, phosphorylation of LARG at S1288 markedly enhances the assembly of the LARG-RhoA complex and subsequent activation of RhoA through GTP loading. Analysis of patient-derived glioblastoma (GBM) cell lines revealed a correlation between RSK activation and LARG S1288 phosphorylation. Moreover, GBM tissue samples showed LARG S1288 phosphorylation and RhoA-GTP–bound RhoA. Elucidating the regulatory mechanisms governing this process is crucial for the development of LARG-targeted therapeutic interventions.

Most cancer deaths are caused by the invasion and metastasis of the original tumor to new organ sites. The mechanisms underlying this process are highly complex and may provide new targets to prevent the spread of highly invasive tumors. Glioblastoma (GBM), the most prevalent and malignant brain tumor, is one such aggressively invasive tumor that has the potential to impact individuals across all age groups. Once diagnosed GBM has a very low survival rate and is characterized by widespread infiltration, resistance to cell death, and uncontrolled cell growth (1). One of the primary challenges hindering the successful management of GBM is the infiltration of neoplastic cells into surrounding normal tissue. These infiltrated cells exhibit heightened resistance to conventional therapeutic modalities like chemotherapy and radiation therapy following surgical intervention, consequently elevating the risk of tumor recurrence (2). The precise molecular mechanisms underlying the aggressive nature of GBM remain unclear. The invasion of solid tumors is known to be modulated by signaling pathways downstream of Rho GTPases (3). RhoA is a member of the Ras superfamily of GTPases and regulates cellular processes such as cell migration, polarization, proliferation, gene transcription, adhesion, and cytoskeletal structure. RhoA functions as a molecular switch that cycles between an active state (GTP bound) and an inactive state (GDP bound). This switching process is mediated by guanine nucleotide exchange factors (GEFs) to promote the exchange of GDP for GTP and requires GTPase-activating proteins (GAPs) to facilitate their intrinsic GTPase enzymatic activity and inactivation (4, 5, 6).

The leukemia-associated RhoGEF (LARG), also known as ARHGEF12, was initially identified as a novel fusion partner of the mixed-lineage leukemia protein in a patient with acute myeloid leukemia (7). LARG, as a GEF, exhibits specificity towards RhoA, but not Rac1 nor Cdc42 (8, 9), facilitating the activation of RhoA by catalyzing the exchange of GDP with GTP (10, 11). The LARG N-terminal domain contains a PDZ (post-synaptic density 95; disk large, zona occludens-1) domain that mediates protein–protein interactions within the target protein (12) and a Regulators of G protein Signaling (RGS) domain that interacts with the small G proteins Gα12 and Gα13, which activate LARG GEF activity (13) (Fig. 1*A*). The LARG C-terminal domain contains a pleckstrin homology (PH) domain, which regulates GEF activity through direct binding with the Rho GTPase and subcellular localization by targeting some RhoGEFs to the cytoskeleton (14, 15, 16). LARG contributes to the development and progression of several diseases, especially cancer, through its ability to activate RhoA (17, 18). Correlations have been identified linking LARG with increased invasion and migration in both GBM cells and brain tissues (10, 11). Moreover, studies have shown that depletion of LARG results in enhanced sensitivity to chemotherapy in GBM-derived cell lines. Phosphorylation of LARG has been observed downstream of various signaling pathways, including G protein-coupled receptors (GPCR), epidermal growth factor receptor (EGFR), and integrin activation, affecting RhoGEF localization and activity (19, 20, 21, 22). Thrombin-mediated activation of GPCRs induces activation of focal adhesion kinase (FAK), which promotes tyrosine phosphorylation in the LARG C-terminal domain at a heretofore unspecified site leading to increased RhoA activation. Deletion of the LARG C-terminal domain inhibits FAK-mediated tyrosine phosphorylation of LARG (23). Integrin activation by mechanotransduction increases LARG phosphorylation through the Src family tyrosine kinase Fyn and promotes its subsequent recruitment to focal adhesion complexes (20). Cyclin-dependent kinase 1 (CDK1) deactivates LARG through phosphorylation at serine 190 in the N-terminal region and serine 1176 in the C-terminal region (24). Whereas p90 Ribosomal S6 kinase 2 (RSK2) activates LARG through phosphorylation at serine 1288 in the C-terminal domain, leading to the activation of RhoA by an unknown mechanism (11). In this study, we describe the mechanism by which phosphorylation at S1288 regulates LARG and its effects on cell invasion, localization, RhoA interaction and oligomerization. Understanding this regulatory mechanism is essential to effectively target these functions therapeutically.

**Figure 1:**
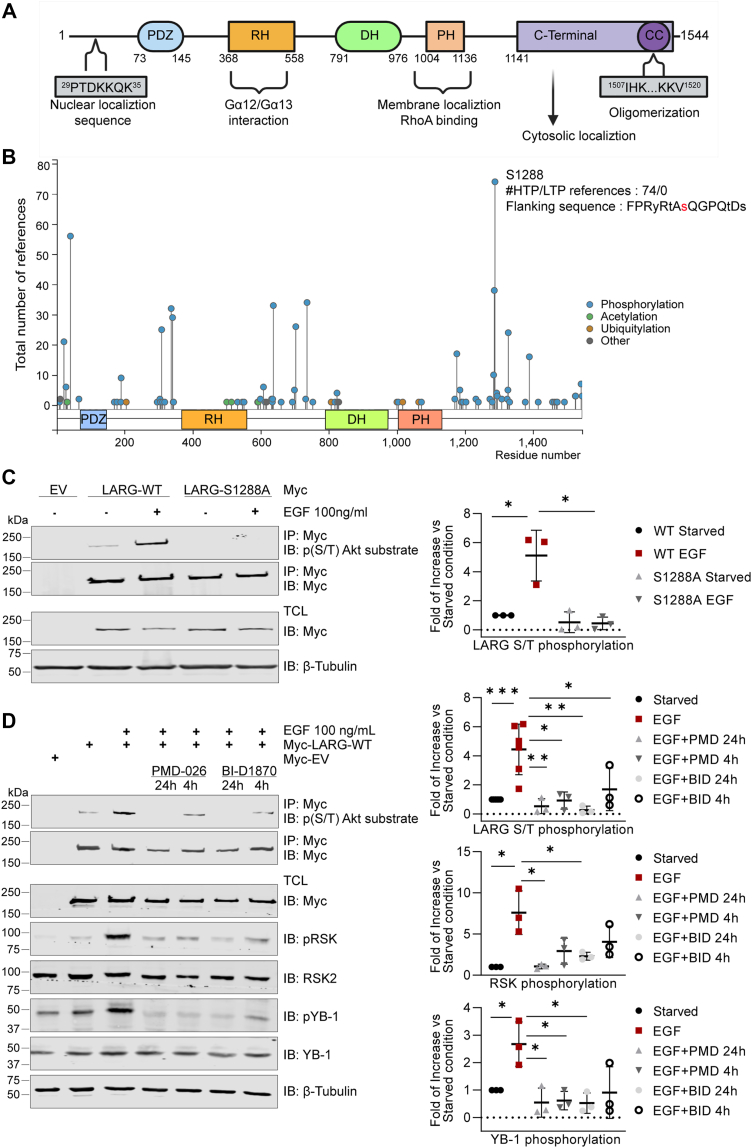
**EGFR activation leads to phosphorylation of LARG at S1288 through RSK**. *A*, schematic representation of of the LARG protein showing the functional domains: PDZ, RH, DH, PH, and CC. These domains coordinate LARG activation, RhoA interaction and localization. *B*, posttranslational modifications determined by Mass Spec are shown as curated by PhosphoSitePlus (www.phosphosite.com). The height of the lines relates to the number of reports in which that modification was identified. *C*, Western blot analysis of LARG phosphorylation from U87 MG cells transfected with Myc-LARG-WT, Myc-LARG-S1288 A, or Myc-EV for 48h. Cells were starved for 24h, then treated with EGF (100 ng/ml) for 10 min. Cell lysates were immunoprecipitated with a Myc antibody, and LARG phosphorylation was detected using a phospho-AKT substrate antibody. LARG phosphorylation was quantified relative to Myc tag expression (*right*). *D*, starved cells were subjected to RSK inhibitors B-ID1780 or PMD-026 for 24h or 4h before treatment with 100 ng/ml EGF for 10 min. LARG, RSK and YB1 phosphorylation levels were quantified as fold changes (*right*). Results are representative of three independent experiments, each of which was done in triplicate and presented as the mean ± standard deviation. ∗*p* ˂ 0.05.

## Results

### EGF stimulation phosphorylates LARG at S1288 through RSK activation

**Figure S1:**
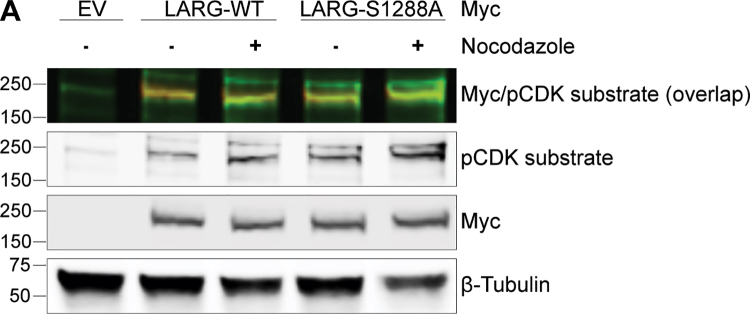


LARG contains an N-terminal PDZ and RH domain that interacts with the small G proteins Gα12 and Gα13, leading to activation of its GEF activity. The C-terminal region comprises DH, PH, and coiled-coil (CC) domains, which mediate RhoA binding, subcellular localization, and oligomerization, respectively (19). LARG Serine 1288 is located in the C-terminal domain and is a frequently phosphorylated residue in many cellular contexts as found in multiple general mass spec analyses spanning 74 studies (PhosphoSitePlus database, www.phosphosite.org). We hypothesize that this reflects the importance of this phosphorylated site in regulating LARG function (Fig. 1*B*). We examined the effect of EGFR activation on LARG phosphorylation. LARG phosphorylation was detected using the Phospho-(Ser/Thr) Akt substrate antibody, which recognizes proteins and peptides containing phosphorylated serine or threonine preceded by arginine residues at positions −5 and −3 (RXRXXS or RXRXXT), a motif also shared by RSK2 substrates (11). LARG contains two recognition sequences RKRNE**T-**858 and RYRTA**S-**1288. As expected from our prior evidence (11), EGF stimulation for 10 min significantly induced LARG-Wild type (WT) S/T phosphorylation and untreated cells maintained a low level of phosphorylation. However, the substitution of serine 1288 with alanine (S1288 A) to prevent phosphorylation at this site impeded the elevation of LARG phosphorylation following EGF stimulation, confirming that EGF stimulation leads to phosphorylation of LARG at S1288 (Fig. 1*C*). RSK inhibitors BI-D1870 and PMD-026 were used to determine whether RSK activation is required to induce the phosphorylation of LARG upon EGF stimulation. The phosphorylation status of endogenous RSK2 was evaluated through immunoblotting using phospo-p90RSK (Ser 380) antibody. YB-1 phosphorylation served as a measure of RSK kinase activity and inhibition, as YB-1 is a downstream target of RSK signaling pathways (25). Treatment with RSK inhibitors notably diminished the effect of EGF on inducing LARG-WT phosphorylation, as cells treated with EGF alone exhibited increased LARG phosphorylation and this was impaired by addition of the RSK inhibitors (Fig. 1*D*). In addition, EGF stimulation led to a notable increase in RSK and YB-1 phosphorylation, whereas treatment with RSK inhibitors resulted in a significant decrease in RSK and YB-1 phosphorylation levels. In addition, to determine whether the S1288A mutant retains the ability to undergo CDK1-mediated phosphorylation, cells overexpressing LARG-WT or LARG-S1288 A were treated with nocodazole to activate CDK1. Using a phospho-CDK substrate motif antibody, we observed increased phosphorylation in both cases, indicating that CDK1-mediated phosphorylation is preserved in the S1288 A mutant and that the effect of the S1288 mutation is specific to RSK-mediated phosphorylation (Fig. S1). These results demonstrate that EGF stimulation induces phosphorylation of LARG at serine 1288 *via* RSK activation. Our further investigation indicates that while the mutant LARG-S1288A loses its ability to be phosphorylated by RSK2 at S1288, the mutant protein maintains normal phosphorylation interactions with other kinases such as CDK1.

### LARG induces cell motility through Ser1288 phosphorylation

**Figure 2:**
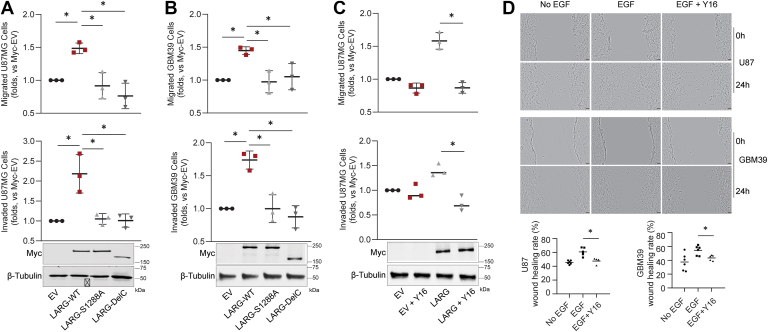
**Phosphorylation at S1288 is required for LARG to induce cell motility**. *A*, U87 MG cells and *B*, GBM39 cells were transfected with LARG-WT, LARG-S1288 A, LARG-delC, or Myc-EV for 48h. Transfected cells were serum-starved before the assay. Cell migration was assessed by Transwell assay (*top*), and cell invasion was assessed by Transwell assay with matrigel (*bottom*). *C*, the LARG-specific inhibitor Y16 (10 μM) was used to test cell migration (*top*) and invasion (*bottom*) of U87 MG cells transfected with Myc-LARG-WT or Myc-EV. *D*, endogenous LARG activity was tested in U87 MG and GBM39 cells using a wound healing assay. The migration rate was calculated as Wound Healing (%) = (Initial wound area - Final wound area)/Initial wound area × 100. Scale bar 100 μm. Results are representative of three independent experiments, each was done in triplicate and presented as the mean ± standard deviation. ∗*p* ˂ 0.05.

To investigate the role of phosphorylated Ser1288 in LARG on cell motility, we overexpressed either LARG-WT or the LARG-S1288A mutant in U87MG GBM-derived cells and assessed their effects on cell migration and invasion using a transwell assay. We used a C-terminal deletion (1141–1544) as a negative control because the C-terminal is known to regulate LARG’s GEF activity (26). Overexpression of LARG-WT in U87MG cells significantly increased cell migration and invasion compared to cells transfected with either an empty vector or the LARG-S1288 A mutant (Fig. 2*A*). Immunoblot analysis confirmed efficient overexpression of LARG proteins. We also overexpressed LARG in the primary GBM PDX cell lines, GBM39, and the effect on cell migration and invasion was examined. Consistent with the results obtained with U87 MG cells, overexpression of LARG-WT in GBM39 resulted in a significant increase of cell migration and invasion relative to cells transfected with either the empty vector or LARG-S1288 A mutant (Fig. 2*B*). To determine if LARG directly induces cell motility, we treated cells with a LARG-specific inhibitor Y16 (27) that blocks LARG binding to RhoA and assessed its impact on motility. The increased migration and invasion observed in U87 MG cells overexpressing LARG-WT were significantly inhibited by Y16 treatment, indicating that LARG can directly regulate cell migration and invasion (Fig. 2*C*). Additionally, we treated U87 MG and GMB39 cells with EGF to induce cell migration as measured by a wound healing assay. This EGF-induced migration was inhibited by Y16, demonstrating that the enhanced migration was due to LARG activity on RhoA, which is effectively blocked by Y16 treatment (Fig. 2*D*).

### Inducing LARG phosphorylation at S1288 by EGF stimulation induces its membrane localization

**Figure 3:**
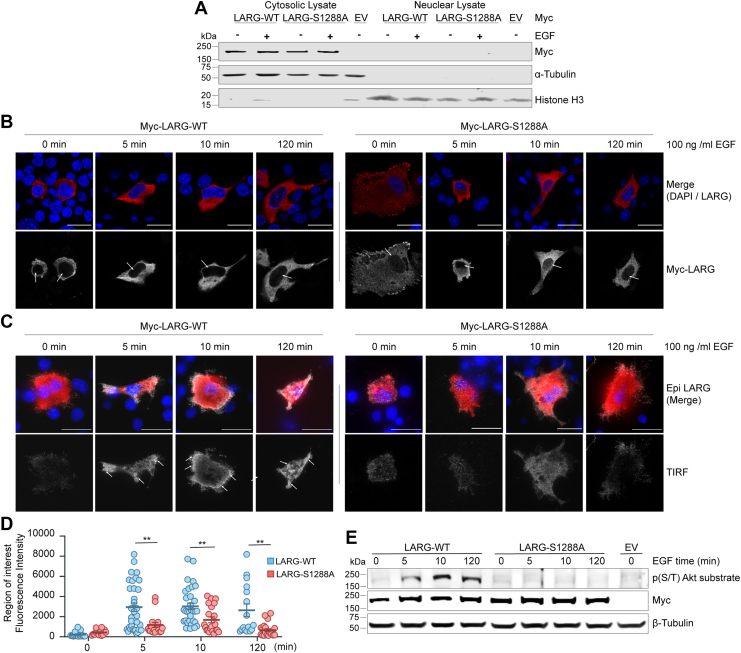
**EGF stimulation induces LARG membrane localization through phosphorylation at S1288**. *A*, U87MG cells were transfected with Myc-LARG-WT, Myc-LARG-S1288 A, or Myc-EV for 48h. Cells were starved for 24h before treatment with 100 ng/ml EGF for 10 min. Nuclear and cytosolic lysates were analyzed to determine LARG localization by immunoblotting with a Myc antibody. Histone H3 and α-tubulin antibodies were used as a control for nuclear and cytosol fractionation respectively. *B–D*, U87 MG cells were transfected with Myc-LARG-WT, Myc-LARG-S1288 A, Myc-LARG-delC, or Myc-EV for 48h. Cells were starved for 24h before treatment with 100 ng/ml EGF for 0- 5-10- and 120-min. Cells were stained with Myc antibody *(red*) and DAPI (*blue*) respectively. *B*, confocal microscope was used to determine the localization of different LARG constructs. *Arrows* indicate nucleus. *C*, total Internal Reflection Fluorescence (TIRF) microscopy was used to visualize Myc-LARG membrane localization. *Arrows* indicate membrane localization. Scale bar 20 μm. All images are representative of at least three independent experiments. *D*, quantification of LARG fluorescence intensity obtained from TIRF microscopy. Each dot represents to an individual cell; ∗∗*p* < 0.01. *E*, Western blot analysis was performed to validate the phosphorylation levels of Myc-LARG-WT and Myc-LARG-S1288 A following EGF stimulation at different incubation times, using a phospho-AKT substrate antibody.

One possible mechanism by which phosphorylation of LARG at S1288 affects its activation of RhoA is by altering LARG sub-cellular localization. Grogač *et al*. reported that LARG can translocate to the nucleus either through disruption of its C-terminal oligomerization domain or *via* a nuclear localization sequence within the N-terminus (26). We examined both nuclear and membrane localization in response to phosphorylation. We utilized two approaches to investigate the impact of phosphorylation on LARG nuclear localization: a subcellular fractionation assay and immunofluorescence staining analyzed by confocal microscopy. EGF stimulation was used to induce LARG phosphorylation at S1288 as previously demonstrated (Fig. 1*B*). Analysis from subcellular fractionation revealed predominant cytoplasmic localization of LARG-WT, with absence of LARG in the nuclear lysate under conditions of serum starvation or EGF stimulation. Furthermore, blocking phosphorylation by mutating LARG S1288 to A1288 showed no significant difference compared to WT (Fig. 3*A*). To validate this finding, LARG nuclear localization was also assessed through immunofluorescence. Results indicated that neither LARG-WT nor LARG-S1288 A exhibited differences in nuclear localization under EGF stimulation or starved conditions (Fig. 3*B*).

However, the confocal imaging revealed that EGF stimulation led to increased membrane localization of LARG-WT, contrasting with non-stimulated cells or stimulated cells transfected with Myc-LARG-S1288A. To more specifically determine membrane localization of LARG we examined membrane localization using Total Internal Reflection Fluorescence (TIRF) Microscopy. EGF stimulation for 5 min induced membrane localization of LARG-WT, persisting for 120 min, whereas LARG-S1288A failed to exhibit a significant increase in membrane localization (Fig. 3, *C* and *D*). The phosphorylation status of Myc-LARG-WT and Myc-LARG-S1288 A following EGF stimulation at varying time points was verified through immunoblotting with phospho(S/T) AKT substrate antibody. EGF stimulation for 5 min was sufficient to induce LARG phosphorylation, with a subsequent increase in phosphorylation levels observed with prolonged stimulation periods (Fig. 3*D*). Taken together, phosphorylation of LARG induces its membrane localization with no effect on nuclear localization.

### LARG phosphorylation at S1288 induces its ability to bind and activate RhoA

**Figure 4:**
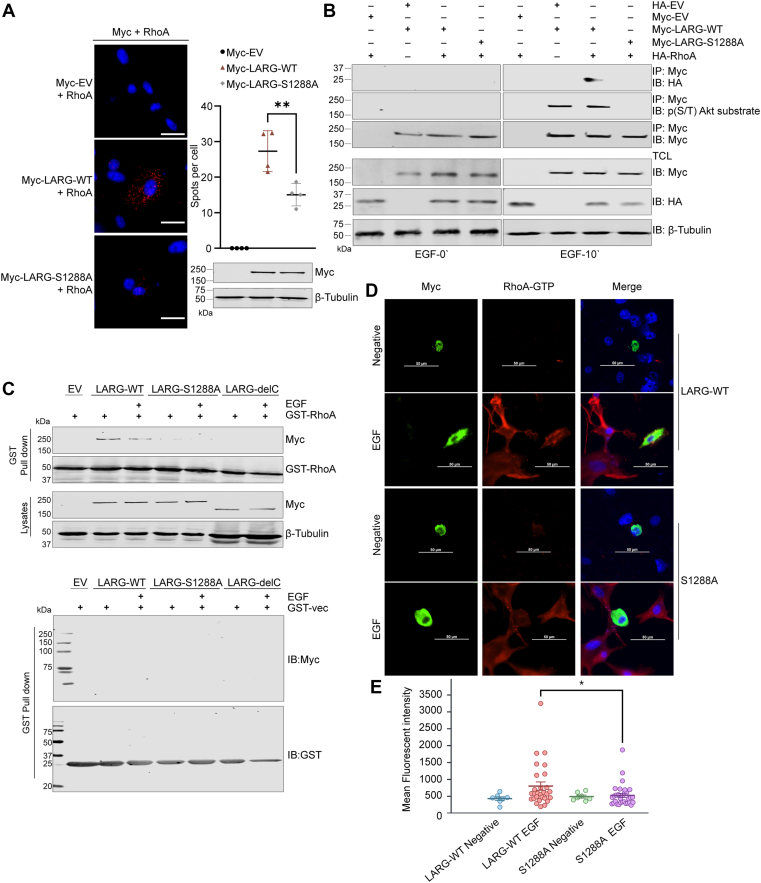
**LARG phosphorylation at S1288 induces its ability to bind and activate RhoA**. *A*, U87MG cells were transfected with Myc-LARG-WT, Myc-LARG-S1288 A, or Myc-EV for 48h. Protein - protein interaction of endogenous RhoA with exogenous LARG-WT or S1288 A mutant was determined by proximity ligation assay. Nuclei were counterstained with DAPI (*blue*). Red fluorescent dots represent PLA signals indicating the interaction between RhoA and Myc-LARG-WT or S1288 A. Expression of Myc-LARG-WT and S1288 A in U87 cells was confirmed by immunoblotting. All images are representative of four independent experiments. Scale bar 20 μm ∗∗*p* < 0.05. *B*, U87 MG cells were co-transfected with Myc-LARG-WT, LARG-S1288 A, or Myc-EV along with HA-RhoA-WT or HA-EV for 48h and starved for 24h, then treated with or without 100 ng/ml EGF for 10 min. The interaction between LARG and RhoA was determined by immunoprecipitating Myc tagged LARG and immunoblotting for HA-RhoA. n = 3. *C*, U87 MG cells were transfected with Myc-LARG-WT, LARG-S1288 A, Myc-LARG-delC, or Myc-EV for 48h and starved for 24h, then treated with 100 ng/ml EGF for 10 min vs nontreated cells. The interaction was assessed by incubating total cell lysates with beads bound to GST-RhoA or GST-vector, followed by immunoblotting for Myc antibody. Results are representative of three independent experiments. *D*, U87 MG cells were transfected with Myc-LARG-WT or Myc-LARG-S1288A for 48h. Cells were then serum-starved for 24h and treated with EGF for 120 min. Cells were then fixed and stained with anti-Myc (*green*) and anti-RhoA-GTP (*red*) antibodies. DAPI was used as a nuclear counterstain (*blue*). Images are representative of two independent experiments. White boxes highlight the RhoA-GTP signal in the LARG-transfected cells. Scale bar 50 μm. *E*, Nikon NIS-Elements software was used to trace the perimeter of all LARG-transfected cells and to quantify the mean cellular fluorescent intensity of the RhoA-GTP signal. Mean cellular fluorescent intensity was graphed for all WT-LARG untreated cells (n = 7), WT-LARG EGF-treated cells (n = 28), LARG-S1288 A untreated cells (n = 7), and LARG-S1288 A EGF-treated cells (n = 31). Statistical significance was assessed *via* two-tailed *t* test (∗*p* < 0.05).

We next investigated the effect of LARG S1288 phosphorylation on the formation of LARG-RhoA complex. A Proximity Ligation Assay (PLA) was used to detect the binding *in situ* between endogenous RhoA and overexpressed Myc-LARG-WT or Myc-LARG-S1288 A in U87 MG cells. PLA analysis revealed an interaction between exogenously expressed LARG-WT and endogenous RhoA. However, the LARG-S1288 A mutant showed a significant reduction in RhoA binding. (Fig. 4*A*). To support this, disruption of LARG-RhoA binding was further validated *via* co-immunoprecipitation using Myc-LARG and HA-RhoA proteins. The LARG-S1288 A mutation, lacking phosphorylation, did not interact with RhoA, whereas the wild-type form of LARG maintained interaction with RhoA (Fig. 4*B*). *In vitro* interaction analysis showed a consistent protein interaction between purified RhoA and LARG-WT, regardless of EGF stimulation, indicating a direct interaction (Fig. 4*C*). In contrast, the LARG-S1288 A mutant and the LARG-delC protein, which lacks the C-terminus (residues 1141–1544) including the S1288 phosphorylation site, disrupted binding with RhoA. These data support the hypothesis that phosphorylation at S1288 is crucial for LARG interaction with RhoA and thereby regulates LARG RhoGEF activity.

We utilized immunofluorescence staining to evaluate the importance of the LARG S1288 phosphorylation site for the activation of RhoA. U87 MG cells were transfected with either the Myc-LARG-WT or the mutant Myc-LARG-S1288A. These cells were then either treated with EGF or incubated in control medium prior to fixation and staining for both Myc-tag and activated RhoA-GTP (Fig. 4*D*). Images were taken and the RhoA-GTP signal was quantified in all Myc-tag-positive cells. We found that the EGF-treated cells demonstrated increased RhoA-GTP signal compared to untreated controls. The EGF-treated cells expressing LARG-S1288 A mutant exhibited less RhoA-GTP staining than the EGF-treated cells transfected with wild-type LARG, as measured by mean cellular fluorescent intensity (Fig. 4*E*). This is further evidence that LARG phosphorylation at S1288 is crucial for its subsequent activation of RhoA and that RSK is a key facilitator of the EGF-mediated activation of RhoA.

### LARG phosphorylation at S1288 does not affect oligomerization

**Figure 5:**
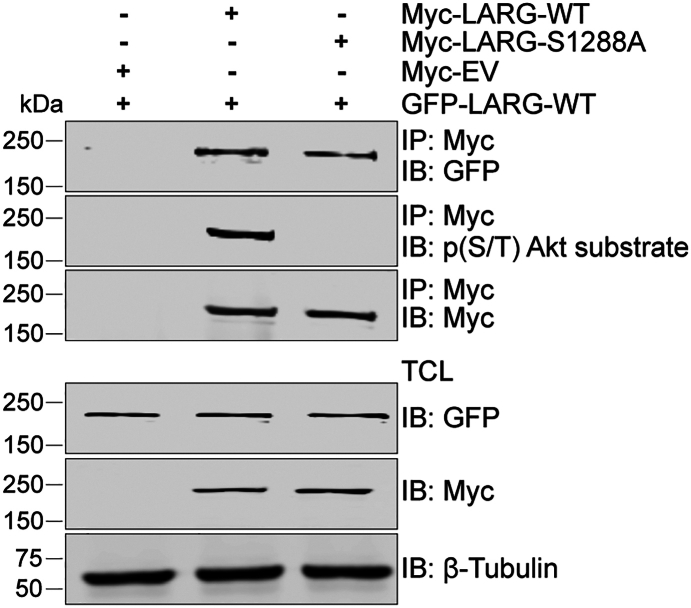
**LARG Phosphorylation at S1288 does not affect its homo-oligomerization**. U87 MG cells were transfected with Myc-LARG-WT, Myc-LARG-S1288 A, or Myc-EV for 24 h, followed by transfection with GFP-LARG-WT. The cells were incubated for an additional 48 h to achieve maximum protein expression. Complex formation was assessed by immunoprecipitating Myc-tagged LARG and performing immunoblotting for GFP. The phosphorylation levels of Myc-LARG-WT and Myc-LARG-S1288 A were confirmed by phospho-AKT substrate antibody. The results are representative of three independent experiments.

We investigated whether phosphorylation of LARG at S1288 influences its homo-oligomerization. The interaction between two different LARG constructs, GFP-LARG-WT and Myc-LARG-WT/S1288 A, was assessed through co-immunoprecipitation to examine homo-oligomerization. The phosphorylation levels of Myc-LARG-WT and Myc-LARG-S1288 A were validated, confirming that LARG-WT was phosphorylated while LARG-S1288 A remained unphosphorylated. Both the phospho-deficient S1288 A mutation and the phosphorylated form of LARG were observed to be co-immunoprecipitated with LARG-WT, suggesting that phosphorylation does not hinder the formation of LARG oligomers (Fig. 5).

### CAAX-mediated membrane translocation of LARG-S1288 A induces binding to RhoA but is insufficient to activate RhoA

**Figure 6:**
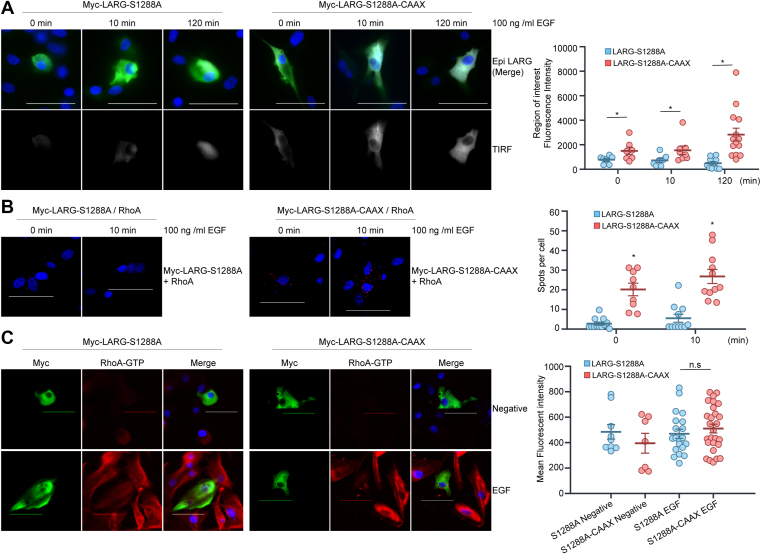
**CAAX-mediated membrane localization of LARG-S1288A increases co-localization with RhoA without affecting RhoA activation**. U87MG cells were transfected with either LARG-S1288A or LARG-S1288A-CAAX to determine whether induced membrane localization of mutant LARG would activate RhoA. *A*, Addition of the CAAX motif to LARG-S1288 A increased membrane localization of protein, particularly in response to EGF stimulation. Transfected cells were serum-starved and treated with EGF for 0, 10 or 120 min. Cells were fixed and stained with anti-Myc (*green*) antibodies. DAPI was used as a nuclear counterstain (*blue*). TIRF imaging was performed to measure membrane localization of Myc-tagged LARG. Images are representative of two independent experiments with either TIRF images alone or merged TIRF/Myc/DAPI images. TIRF signal intensity for each Myc-tagged cell measured was graphed, and statistical significance between LARG-S1288 A and LARG-S1288A-CAAX was assessed for each time-point *via* two-tailed *t* test (∗*p* < 0.05). *B*, LARG-S1288A-CAAX demonstrates increased co-localization with RhoA compared to LARG-S1288A. Transfected U87 MG cells were serum-starved and treated with EGF for 0 or 10 min, at which point they were fixed and stained with anti-Myc and anti-RhoA antibodies. Proximity ligation assay was used to visualize interactions between RhoA and Myc-tagged LARG-S1288 A or LARG-S1288A-CAAX, with red fluorescent dots marking points of interaction. Images are representative of two independent experiments. Scale bar 50 μm. Fluorescent dots per cell were quantified and graphed. Statistical significance between LARG-S1288 A and LARG-S1288A-CAAX was assessed for each time-point *via* two-tailed *t* test (∗*p* < 0.05). *C*, Cells transfected with LARG-S1288A-CAAX show no increase in active RhoA-GTP expression compared to LARG-S1288A. Transfected U87 MG cells were serum-starved, then either untreated or treated with EGF 120 min. Cells were then fixed and stained with anti-Myc (*green*) and anti-RhoA-GTP (*red*) antibodies. DAPI was used as a nuclear counterstain (*blue*). Images are representative of two independent experiments. Scale bar 50 μm. Nikon NIS-Elements software was used to trace the perimeter of all LARG-transfected cells and to quantify the mean cellular fluorescent intensity of the RhoA-GTP signal. Mean fluorescent intensity of RhoA-GTP was graphed for each Myc-positive cell imaged. Statistical significance between EGF-treated and untreated cells was assessed *via* two-tailed *t* test (*p* < 0.05).

To determine whether the inability of LARG-S1288 A to activate RhoA was due only to the protein’s impaired translocation to the cellular membrane or to a secondary effect on LARG-RhoA interactions, we utilized an HRAS CAAX motif that would facilitate membrane trafficking of LARG-S1288 A (28). TIRF imaging confirmed that the LARG-S1288A-CAAX protein translocated to the cell membrane more than LARG-S1288A, and that this was further enhanced in response to EGF stimulation (Fig. 6*A*). The extent of the EGF-dependent effect of LARG-S1288A-CAAX cell membrane localization was unexpected. However, the HRAS CAAX sequence is known to induce localization not only to the cell membrane but also to other membranous components of the exocytic pathway, particularly the Golgi apparatus (28). Noting this, it may be that the LARG-S1288A-CAAX protein localizes to various membranous compartments within the cell under basal conditions before trafficking primarily to the cell membrane upon the introduction of the secondary signal of EGF stimulation. Even so, PLA analysis indicated that the LARG-S1288A-CAAX protein was able to complex with RhoA to a greater extent than LARG-S1288A under both basal and EGF-stimulated conditions (Fig. 6*B*). However, despite the increase in LARG membrane localization and RhoA interactions, ICF imaging demonstrated no increase in activated RhoA-GTP expression in LARG-S1288A-CAAX transfected cells *versus* LARG-S1288A under EGF stimulation (Fig. 6*C*). This may indicate that phosphorylation at LARG S1288 affects both the protein’s membrane localization and, separately, its GEF activity, or it may be that the addition of the CAAX motif on the C-terminal domain of LARG interferes with its capacity to activate RhoA.

### Endogenous LARG phosphorylation at S1288 and downstream activation in GBM tissues

**Figure 7:**
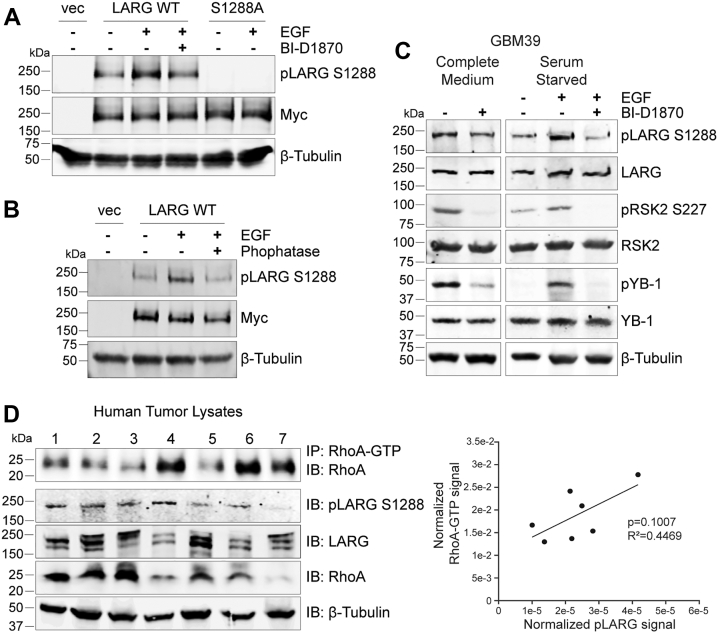
**Endogenous LARG phosphorylation at S1288 and RhoA activation in GBM tissues**. *A*, the phospho-LARG S1288 specific antibody was tested in U87MG cells transfected with Myc-LARG-WT, Myc-LARG-S1288 A, or Myc-EV. After 24 h of starvation, cells were treated with EGF for 10 min in the presence or absence of BI-D1870. *B*, the phospho-LARG S1288 antibody demonstrates specificity to the phosphorylated form of the protein. U87MG cells were transfected with Myc-LARG-WT of Myc-EV. After 24 h of starvation, cells were treated with EGF for 10 min. Protein was harvested and lysates were treated with λ phosphatase prior to immunoblotting. *C*, RSK2 phosphorylates LARG at S1288 in PDX-derived GBM39 cells. Cells maintained in complete medium with 10% FBS were treated with BI-D1870 for 4 h. Subsequently cells were starved in 0.1% FBS medium for 24 h prior to 10 min EGF treatment alone or after prior 4 h BID treatment. Immunoblotting was used to determine total expression and phosphorylation of target proteins. The results are representative of three independent experiments. *D*, levels of activated RhoA-GTP positively correlate with levels of phosphorylated LARG S1288 in human GBM tumor lysates. Endogenous total LARG, pLARG-S1288 and RhoA expression in seven human GBM tumor lysates were analyzed by immunoblotting. Activated RhoA-GTP was isolated by immunoprecipitation and detected with a RhoA antibody. Signals for each target protein expression were normalized to corresponding sample β-tubulin expression. A linear regression analysis was performed to assess the relationship between Activated RhoA expression and pLARG S1288 expression in all seven tumors.

To investigate the status of endogenous LARG phosphorylation at S1288 in human GBM tissues, we developed a customized antibody specific for detecting phospho-LARG S1288. To validate the antibody, U87 MG cells were transfected with Myc-LARG-WT and Myc-LARG-S1288 A, followed by EGF stimulation and BI-D1870 treatment to block RSK activity. The phospho-LARG S1288 antibody showed a clear increase following EGF stimulation in LARG-WT, while this was inhibited by BI-D1870 treatment or LARG-S1288 A mutation (Fig. 7*A*). To further assess phosphorylation specificity, cells were stimulated with EGF and then treated with phosphatase, which resulted in a decrease in the phosphorylation signal (Fig. 7*B*). Following antibody validation, we examined the GBM patient-derived xenograft cell line, GBM39, to detect phospho-LARG S1288. First, cells in complete medium supplemented with 10% FBS were treated with the RSK inhibitor BI-D1870. This led to the reduction in autophosphorylation of RSK2 at S227, as well as a reduction in LARG phosphorylation at S1288 and in phosphorylation of the downstream RSK target YB1. Subsequently, GBM39 cells were serum starved in 0.1% FBS medium prior to treatment with EGF alone or following pretreatment with BI-D1870. Activation of RSK with EGF resulted in an increase in phosphorylation in RSK2, LARG and YB1. However, pretreatment with BI-D1870 largely attenuated the observed EGF effect in all three targets (Fig. 7*C*). These results provide evidence that RSK activity regulates LARG at S1288 in a GBM patient-derived xenograft.

To further explore LARG phosphorylation in GBM samples, western blots were performed on human GBM tumor lysates. Immunoblotting revealed varying levels of endogenous expression of total LARG, pLARG S1288 and RhoA between the tumor samples. Because the epitope recognized by our RhoA-GTP antibody is disrupted by SDS, it can be used for immunoprecipitation but not for Western blot. Therefore, to quantify activated RhoA in the tumor lysates, we used the RhoA-GTP antibody to pull down activated RhoA from the samples, which we could then visualize and quantify with a RhoA antibody. Interestingly, both activated RhoA-GTP and pLARG S1288 were expressed, and this limited correlation, although not statistically significant, was stronger than that observed between RhoA-GTP and endogenous RhoA or between RhoA-GTP and total LARG (Fig. 7*D*). This suggests that LARG phosphorylated at S1288 can play a role in the activation of RhoA in human GBM tumors.

## Discussion

**Figure 8:**
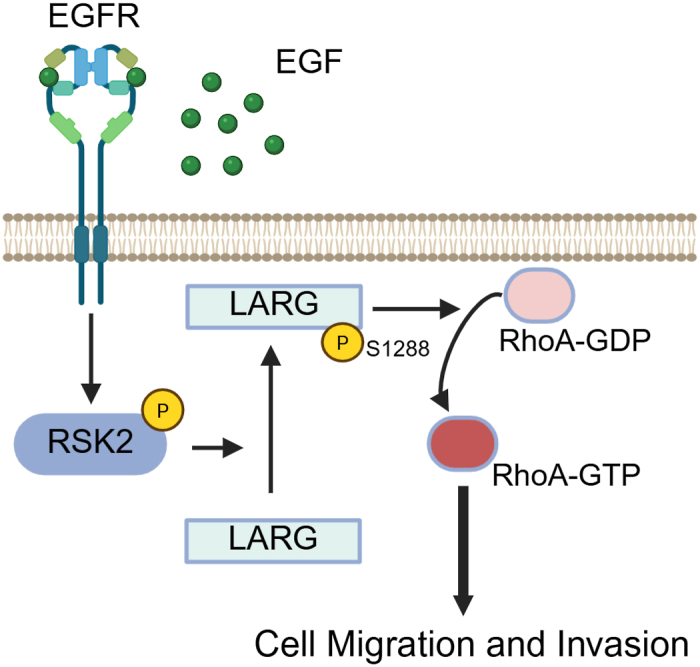
**Model of LARG activation of RhoA in response to RSK2 phosphorylation**. Schematic of the proposed signaling mechanism in which EGF stimulation activates RSK2, which leads to the phosphorylation of LARG at serine 1288. Phosphorylation of LARG at serine 1288 then promotes plasma membrane localization of LARG and binding to RhoA and subsequent RhoA activation.

Human cancer development and progression have been linked to the activation of RhoA, which is facilitated by GEFs such as LARG, resulting in its effect on invasion and migration (29, 30). When LARG is deleted in cancer cells, RhoA activation is significantly reduced, and invasion and migration are impeded (11). LARG, therefore, contributes to the regulation of cell invasion, proliferation, migration, and metastasis by catalyzing the exchange of GDP for GTP on RhoA, thereby activating RhoA and its downstream pathways. In this study, we demonstrated that LARG protein expression significantly enhances invasion in GBM-derived cell lines through phosphorylation at S1288 in response to EGF stimulation. LARG has been shown to be activated upon EGFR, GPCR, and integrin activation (11, 20, 31, 32). Epidermal growth factor receptor (EGFR) is one of the major targets for GBM treatment, and it is correlated with increasing glioma invasion (33). Our proposed signaling mechanism posits that EGF stimulation activates RSK2, which leads to the activation and membrane localization of LARG, as well as the subsequent activation of RhoA (Fig. 8). Our results showed that EGF stimulation increased LARG phosphorylation at S1288, while phosphorylation was reduced in mutant LARG-S1288 A compared to LARG-WT. The phosphorylation of LARG-WT was significantly reduced by RSK inhibitors under EGF stimulation, indicating that EGFR signaling leads the phosphorylation of LARG at S1288 through RSK activation. Additionally, we demonstrated RSK-dependent phosphorylation of LARG at S1288 in a PDX cell line as well as a positive correlation between pLARG S1288 and activated RhoA-GTP expression in primary tumor samples, indicating that this pathway is conserved in patient GBM tissue as well as in established cell lines.

Several conserved regions are present in the C-terminal domain of LARG, including a pleckstrin homology (PH) domain and a Dbl homology (DH) domain, which is responsible for its GEF activity (4). The DH domain of LARG features a unique N-terminal extension consisting of two short helices (residues 733–781) that are essential for its GEF activity and directly interact with RhoA. RhoA specificity is primarily determined by structural elements within the DH domain. The PH domain has been proposed to play a critical role in stabilizing the active conformational structure that facilitates RhoA binding. Additionally, the isolated DH or DH/PH domain of LARG functioned as a strong activator of RhoA (9). Mutations at Ser-1118 and Glu-1023 in the PH domain significantly reduce guanine exchange activity (4). In this study, we demonstrated that LARG phosphorylation at S1288 affects its ability to bind RhoA. Blocking LARG phosphorylation by the substitution of Ser 1288 to Ala significantly reduces LARG-RhoA complex formation, indicating that LARG-S1288 phosphorylation plays an important role in RhoA activation by facilitating its binding to RhoA. S1288 is located at the LARG C-terminal but not in the catalytic domains of LARG (DH and PH domains). The PH domain in particular is indicated to play a major role in recruiting GEFs to the plasma membrane. This suggests that S1288 phosphorylation might mediate LARG-RhoA binding through interacting with other domains like the PH domain of LARG, engaging other regulatory proteins, or increasing the accessibility of LARG's binding site for RhoA. For example, phosphorylation at serine 1288 may regulate accessibility of the PH domain for membrane translocation.

Disrupting the binding between LARG and RhoA may serve as a novel therapeutic approach to inhibiting invasion and migration. A number of inhibitors targeting GEFs, including LARG, have been developed (27, 34). LARG activation of RhoA has been found to be dose dependently suppressed by Y16, which binds to the DH-PH domain junction of LARG inhibiting the binding between LARG and RhoA. Targeting LARG with Y16 in neuroblastoma has been shown to promote differentiation, induce MYCN degradation, and reduce tumorigenicity, suggesting a translatable therapeutic approach for targeting LARG (35). We also showed that treatment with Y16 significantly reduces the migration and invasion capabilities of GBM cells. This finding supports the potential therapeutic value of targeting LARG activity in GBM, as inhibiting these processes may help limit tumor invasion. However, LARG has also been identified as a potential tumor suppressor in human breast and colorectal cancers by reducing colony formation, cell proliferation, and cell migration (36). Future studies are needed to explore the role of LARG in various cancer types and its impact on tumorigenesis.

LARG's C-terminal domain is crucial for the localization and oligomerization of LARG. A coiled-coil sequence located at the end of the C-terminus of LARG (^1507^IHKIEADLEHLKKV^1520^) plays a key role in maintaining LARG in the cytoplasm and controlling LARG oligomerization while LARG N-terminus nuclear localization sequence (^29^PTDKKQK^35^) is responsible for its nuclear import (26). LARG oligomers form through its C-terminal coiled-coil domain, and the substitution of isoleucines 1507 and 1510 with alanines disrupted LARG oligomerization. This disruption of oligomerization did not affect its GEF activity. Our results showed that EGF stimulation induces LARG phosphorylation at S1288 through RSK activation and induces LARG membrane localization. It may be that phosphorylation of LARG at S1288 induces a structural alteration that promotes the protein’s own translocalization to the cell membrane. Alternatively, phosphorylation at S1288 may increase the binding affinity of LARG to a chaperone or scaffolding protein that then facilitates membrane localization. In addition, LARG localization might be regulated by the presence of the LARG C-terminus, LARG N-terminus nuclear localization sequence (^29^PTDKKQK^35^), and stimulate LARG activation.

Blocking the phosphorylation of LARG using the LARG-S1288 A mutant has no significant effect on LARG oligomerization. This finding suggests that LARG oligomerization and its GEF activity are independently regulated through distinct mechanisms within the C-terminal region. However, phosphorylation of GFP-LARG-WT was not detected when co-expressed with the Myc-LARG-S1288 A mutant, raising the possibility that the S1288 A mutation interferes with WT phosphorylation during oligomerization. Further investigation will be needed to clarify this possibility. The addition of an HRAS CAAX motif to target the mutant LARG-S1288 A protein to the plasma membrane did not increase expression of activated RhoA-GTP despite increasing membrane localization and LARG-RhoA interaction. This suggests that the C-terminal region does not block RhoA binding in the LARG-S1288 A mutant and that phosphorylation at S1288 is required not only for membrane localization but also separately for LARG’s ability to activate RhoA. Alternatively, it may be that the addition of the CAAX motif to the C-terminal domain of LARG inhibits the protein’s GEF activity. Further studies on the GEF activity of LARG-WT, LARG-S1288 A and LARG-S1288A-CAAX are needed to explore these possibilities.

In summary, we identified that RSK2 phosphorylation of LARG at S1288 promotes LARG membrane localization and enhances its interaction and activation of RhoA. This provides the mechanism for the previously identified ability of LARG to mediate RSK-driven motility and activation of RhoA. These findings suggest that targeting this pathway could serve as a potential therapeutic strategy to limit cancer cell invasion.

## Experimental procedures

### Plasmids and reagents

Myc tagged LARG-WT was previously made by subcloning using GFP-LARG-WT construct which was kindly provided by Philip B. Wedegaertner, Thomas Jefferson University, Philadelphia. The Myc-LARG-S1288 A mutant plasmid was generated using Quick Change Site-Directed Mutagenesis Kit (Aligent, CA, USA) according to manufacturer's instructions. Primers with forward 5′ AGATACAGAACAGCC**GCT**CAGGGGCCGCAGACA 3′, reverse: 5′ TGTCTGCGGCCCCTG**AGC**GGCTGTTCTGTATCT 3′ were used. Myc-LARG with deleted C-terminal (1141–1544) (Myc-LARG-delC) was made using standard molecular cloning methods. LARG with deleted C- terminal was amplified using PCMV-Myc-LARG-WT as the DNA template, forward Primer 5′ ACCCGTGGATCCGGCGTCATGAGTGGCACACAGTCTACTATC 3′ and reverse primer 5′ CGTCTCGAGCTACTTTGTGGATTGCTC 3′ with 65 °C annealing temperature for 30 cycles. The PCR product was validated by gel electrophoresis and purified from the gel using Qiagen QIAquick Gel Extraction Kit. The purified PCR product (insert) was digested for 4h at 37 °C with BamHI and XhoI restriction enzymes. PcDNA3.1 plasmid (vector) was also digested with BamHI and XhoI for 1h at 37 °C. Digested insert and vector were purified from the gel and ligated overnight at 4 °C at a 1:3 ligation ratio. The ligation mix was then transformed into DH10 B competent *Escherichia coli* cells. The deletion of the C- terminal was validated by sanger sequencing and the expression of the protein with Myc tag was validated by Western blot. The Myc-LARG-S1288A-CAAX plasmid was generated from the Myc-LARG-S1288 A backbone by adding a C-terminal CAAX sequence 5′ TGCATGAGCTGCAAGTGTGTGCTCTCC 3’. The modified construct was synthesized and sequence-verified using the GeneArt Gene Synthesis Service (Thermo Fisher Scientific). Myc tag (9B11) mouse, Myc tag (71D10) rabbit, Phospho-(Ser/Thr) Akt Substrate (9611) rabbit, phospho-p90RSK (Ser380) (D3H11) rabbit, YB1 (D299) rabbit, phospho-YB1 (Ser102) (C34A2) rabbit, RhoA (67B9) rabbit, HA-tag biotinylated (C29F4) rabbit, GFP (D5.1) (2956) rabbit, phospho-CDK substrate motif [(K/H)pSP], and histone H3 (D1H2) XP rabbit antibodies were purchased from Cell Signaling Technology. RhoA (sc-418) mouse, α tubulin (sc-8035) mouse, YB1 (sc-101198) mouse, RSK2 (sc-9986) mouse, LARG H-3 (sc-166318) mouse, GST (sc-374171) mouse monoclonal antibodies, and RSK2 inhibitor BI-D1870 were purchased from Santa Cruz Biotechnology. RSK2 inhibitor PMD-026 was a gift from Sandra Dunn (Phoenix Molecular Designs, Vancouver, BC, Canada). Myc tag (16286-1-AP) rabbit, RSK2 (23762-1-AP) rabbit, β tubulin (66240-1-Ig) mouse antibodies were purchased from Proteintech. Phospho-RSK2 (Ser227) rabbit (ab75820) monoclonal antibody was purchased from Abcam. The RhoA-GTP mouse (26,904) monoclonal antibody was purchased from NewEast Biosciences. Nocodazole was purchased from Millipore Sigma. The anti-phospho-LARG (Ser1288) polyclonal antibody was generated in rabbits using the phospho-peptide Cys-FPRYRTA(pS)QGPQTDS, obtained from Biomatik. The rabbits were immunized with the synthesized peptide, and the antibody was purified through peptide antigen affinity chromatography. Specificity of the anti-phospho-LARG (Ser1288) antibody was confirmed by treating 25 μg protein lysates with 200 units lambda phosphatase sc-200312 (Santa Cruz Biotechnology) and incubating the samples at 30 °C for 5 min prior to immunoblotting.

### Cell culture and transfections

Human GBM U87 MG cells were obtained from the National Cancer Institute’s Tumor Repository and the cell line identity was validated by STR profiling by ATCC and tested negative for *mycoplasma* (LT07–188; Lonza). GBM39 primary GBM patient-derived xenografts (PDXs) were obtained from the Mayo Clinic Brain SPORE (37). Details are available from Mayo Clinic (https://www.mayo.edu/research/labs/translational-neuro-oncology/mayo-clinic-brain-tumor-patientderived-xenograft-national-resource/overview). Cells were maintained in Dulbecco's Modified Eagle Medium (DMEM) supplemented with 10% (vol/vol) fetal bovine serum (FBS), 1% penicillin-streptomycin at 37 °C at 5% CO_2_. Cells were plated in a 100 mm dish or 6-well plate 24h before transfection. Cells were transfected with either 1ug of plasmid DNA per well of 6-well plate or 4ug of DNA in 100 mm dish using X-tremeGENE HP DNA transfection reagent (Sigma-Aldrich, Darmstadt, Germany) according to manufacturer’s protocol for 48h to 72h. Cells were starved with 0.1% FBS for 24h before being subjected to any treatment.

### Western blotting

Cells were washed with phosphate-buffered saline (PBS) and lysed in RIPA buffer (Thermo Fisher Scientific) supplemented with a protease inhibitor cocktail (Roche). Protein lysates were mixed with 4 × SDS sample buffer, boiled for 5 min, and resolved on 4 to 20% Mini-PROTEAN TGX gels (Bio-Rad). Separated proteins were transferred to nitrocellulose membranes, which were blocked with 5% nonfat dry milk in Tris-buffered saline containing 0.1% Tween-20 (TBST) for 1 h at room temperature. Membranes were incubated overnight at 4 °C with indicated primary antibodies, washed three times with TBST, and then incubated with fluorescently labeled secondary antibodies for 1 h at room temperature. After three additional washes with TBST, protein bands were visualized using a LI-COR Odyssey imaging system, and band intensities were quantified using ImageJ software.

### Primary GBM tumor analysis

Seven independent surgical specimens of GBM tumor tissues were obtained from Audubon Bioscience (New Orleans, LA). A table detailing potentially consequential genetic variations between the primary human tumors is available in the supplementary materials (Table S1). The tissues were homogenized in RIPA buffer and sonicated on ice two times for 5 s each with intervals between sonication. The resulting tissue lysates were immunoprecipitated with RhoA-GTP antibody specific for active RhoA and immunoblotted using described antibodies. Standard western blots were used to deternmine expression of total LARG, pLARG-S1288 A, and total RhoA. As the epitope recognized by the RhoA-GTP antibody (NewEast Biosciences) is disrupted by SDS, it was instead used for immunoprecipitation but not western blotting. Therefore, to visualize and determine activated RhoA expression in our samples, the RhoA-GTP antibody was used to pull down the activated form of the protein from the tumor lysates, and this pulled down protein was subsequently immunoblotted for total RhoA. Expression of each target protein was normalized to β-tubulin, and a linear regression analysis was performed to assess the relationship between Activated RhoA expression and pLARG S1288 expression across all seven tumors.

### Intracellular protein-protein interaction analysis

Immunoprecipitation (IP) was used to analyze intracellular protein-protein interactions. U87 MG cells were co-transfected with Myc Myc-tagged form of LARG-WT, LARG-S1288 A, or LARG-delC along with HA-RhoA-WT or GFP-LARG-WT for 48h or 72h as indicated. Cells were starved with 0.1% FBS for 24h before treatment. Cells were lysed at 4 °C using kinase lysis buffer containing 20 mM Hepes (pH 7.4), 150 mM NaCl, 50 mM kF, 50 mM β-glycerolphosphate, 2 mM EGTA (pH 8.0), 1 mM Na3VO4, 1 × protease inhibitor cocktail, 1% Triton X-100 and 10% glycerol. For IP 2 mg of lysate was incubated with 20ul of protein a g plus-agarose sc-2003 (Santa Cruz Biotechnology) together with 0.6 μg of Myc antibody in 1 ml of the final volume for 2h at 4 °C. For tumor lysates, 1 mg of lysate was incubated with 30 μl of protein a g plus-agarose beads with 1.5 μg RhoA-GTP antibody in 1 ml total volume overnight at 4 °C. Then the beads were washed twice with PBS supplemented with protease and phosphatase inhibitors. A protein loading buffer was used to release the proteins from the beads. Proteins were resolved on SDS/PAGE and transferred to nitrocellulose membrane. The interaction was detected by immunoblotting with a specific antibody to the tag fused to the protein of interest, while the proteins of interest expression levels were also determined. The immunoblotting signal was detected using a LI-COR scan.

### In vitro protein-protein interaction analysis

The *in vitro* association analysis was done using purified recombinant GST–fused RhoA-WT protein and Myc tagged form of LARG-WT and mutants. To determine the effect of LARG-S1288 phosphorylation on LARG-RhoA complex formation, U87MG cells were transfected with Myc-LARG-WT, Myc-LARG-S1288 A, Myc-LARG-delC, or Myc-EV as control for 48h to allow maximum protein expression. Cells were starved with 0.1% FBS for 24h before EGF (100 ng/ml) stimulation for 10 min. The cell lysates were prepared using kinase lysis buffer. Then 0.5 mg of the lysate was incubated with 10 μg of glutathione agarose bound to GST-RhoA or GST-alone for control in 1 ml final volume at 4 °C for 2 h with end-to-end rotation. The resins were washed 3 times using kinase lysis buffer, kinase lysis buffer containing 0.5 M NaCl, and kinase lysis buffer, respectively. A protein loading buffer was used to release the proteins from the beads. Proteins were resolved on SDS/PAGE and transferred to nitrocellulose membrane. The interaction was detected by immunoblotting with Myc and GST antibodies. while the proteins of interest expression levels were also determined. The immunoblotting signal was detected using a LI-COR scan.

### Transwell assays

Migration assay was performed using ThinCerts (TC) insert, pore size 8 μm (Greiner Bio-one) and invasion assay was performed using inserts coated with Matrigel (Corning Life Sciences). U87 MG cells were transfected with 1ug of LARG-WT, LARG-S1288 A, LARG-DelC, or Myc-EV for 48h. Then, cells were seeded into the insert at a density of 2 x 10^5^ in 0.2 ml DMEM supplemented with 0.1% FBS. Each insert was placed into a well (24 well plate) containing 0.5 ml of DMEM with 10% FBS. Cells were allowed to invade for 24h in an incubator with 5% CO2 at 37 °C. Non-invaded cells were removed by cotton swab, and inserts were washed twice with DPBS. Inserts were placed into a 24-well plate containing 0.4 ml DMEM no phenol red containing 8 μM Calcein-AM (Sigma-Aldrich) and incubated for 45 min at 37 °C. Then, inserts were washed twice with DPBS and incubated with 0.5 ml trypsin-EDTA (0.5%), no phenol red for 10 min at 37 °C. Invaded cells were quantified in triplicate by measuring fluorescence at excitation wavelength 458 nm and emission wavelength 520 nm. To calculate the increase of fold, the measurement of invaded cells was divided into testing samples by that in the control sample (Myc-EV). To gain significant statistical difference, three separate experiments were performed, and three inserts were used for each group.

### Wound-healing assays

U87 MG cells and Patient-derived GBM cells were allowed to grow to confluence in 6-well culture plates. The cells starved overnight in 0.1% FBS media. Cells were scratched with a 200 μl pipette tip and washed in serum free media and replaced with conditioned media. Cells were allowed to migrate into scratch for 24 h. Images of the scratch were acquired immediately after scratching (t = 0) and at 24 h. The average closure of the scratch by migrating cells was measured using ImageJ software.

### Recombinant protein expression and purification

Bacterial expression vector (pGEX-4T-1-GST-RhoA-WT) was transformed into *E*. *coli* strain BL21 cells (New England BioLabs Inc) and grown in prewarmed LB/amp medium at 37 °C and 275 rpm with vigorous shaking to OD600 of 0.4 to 0.6. 100 uM isopropyl β-D-1-thiogalactopyranoside (IPTG) was added to induce the expression of recombinant protein and incubated for 3h with vigorous shaking at RT. The cells were harvested by centrifuging at 4 °C, 6000 rpm for 10 min. The cell pellet was resuspended with 4 ml TES buffer (50 mM Tris-HCl pH 7.5, 40 mM EDTA pH 8, 25% sucrose). The cells were incubated with 1 ml of 20 mg/ml lysozyme for 1h on ice then 4 ml of GST lysis buffer A (10 mM Tris-HCl pH 7.5, 1 mM DTT, 1 mM PMSF), 10 ml of GST lysis buffer B (20 mM Hepes pH7.6, 100 mM KCl, 1 mM DTT, 1 mM PSMF) and 2 ml of 10% Triton X-100 were added then mixed well. Cells were sonicated on ice for 7-s bursts two times and then centrifuged for 30 min at 4 °C and 10000 x g. Cell extracts were then incubated with glutathione agarose beads at 4 °C for 3h with end-to-end rotation. The beads with bound protein were recovered by centrifugation at 500 x g for 2 min at 4 °C and extensively washed once with GST lysis buffer B, once with GST lysis buffer B supplemented with 1M sodium chloride and once again with GST lysis buffer B. The beads with bound protein were resuspended in GST lysis buffer B and aliquots were stored at −80 °C.

### In Situ proximity ligation assay

U87 MG cells were seeded on six well plate 24h before transfection with Myc-LARG-WT and Myc-LARG-S1288 A mutant construct. Cells were detached 48h after transfection and seeded on an 8-well slide and incubated for 24h. In Situ Proximity Ligation Assay (Sigma-Aldrich, Darmstadt, Germany) was performed according to the instructions provided by the manufacturer. Briefly, cells were fixed with 3.7% formaldehyde for 15 min at room temperature (RT) and washed three times 5 min each with cold PBS. Cells were then permeabilized for 10 min at RT with 0.05% Triton X-100 and blocked in a humidity chamber for 1 h at 37 °C using Duolink *in situ* blocking solution. The eight-well slide was then incubated with Myc tag mouse and RhoA rabbit antibodies overnight at 37 °C in a humidity chamber. The slide was incubated for 1 h at 37 °C with anti-mouse probe and anti-rabbit probe, then washed twice with Duolink wash buffer A. Ligation solution was prepared and added to each sample, then incubated at 37 °C for 30 min. The slide was then washed twice with Duolink wash buffer A and protected from light, and then subjected to polymerase amplification, incubated overnight at 37 °C in a humidified chamber. The slide was washed with Duolink wash buffer B twice, then stained with DAPI. ZEISS Axiovert 200 M microscope was used for imaging, and the red dots were counted using ImageJ software. The transfected Myc-LARG-WT and S1288A mutant expressions were confirmed by western blotting. To ensure the reproducibility of results, the experiment was performed four times independently. The Student’s *t* test was used for the statistical significance of differences between biological replicates.

### Immunofluorescence and cell imaging

U87 MG cells were seeded on coverslips coated with 10 ug/ml fibronectin to enhance cell adhesion. Cells were transfected with the Myc tagged form of LARG-WT, LARG-S1288A, LARG-DelC, or Myc-EV for control for 48h. Cells were starved for 24h before treatment with 100 ng/ml EGF, then fixed for 15 min with 3.7% formaldehyde at room temperature (RT). Then, cells were washed three times with PBS and permeabilized using 0.05% Triton X-100 for 10 min at RT. Cells were blocked in blocking buffer containing 1X PBS, 5% normal serum, 0.3% Triton X-100 for 1 h at RT then incubated overnight with Myc tag and RhoA-GTP antibody. Cells were washed three times with PBS and incubated with Alexa Fluor 488 goat anti-rabbit, and Alexa Fluor 568 goat anti-mouse (Thermo Fisher Scientific) for 1.5 h at RT in the dark. Samples were washed three times with PBS and mounted with mountain media containing DAPI (Ibidi). Epifluorescence images were acquired with a Nikon Eclipse Ts2 microscope (Nikon, Tokyo, Japan). Mean cellular fluorescent intensity for RhoGTP signal for each transfected cell was assessed on NIS-Elements software (Nikon, Tokyo, Japan). Confocal images were acquired on a Leica TCS SP5 confocal microscope (Leica, Wetzlar, Germany) and Total Internal Reflection Fluorescence Microscopy (TIRF) was performed on a Leica Thunder Live Cell 3D microscope with TIRF module (Leica, Wetzlar, Germany) at the University of Hawaii Cancer Center Imaging Core and the Pennington Biomedical Research Center. LARG fluorescence intensity in TIRF microscopy images was measured and quantified using ImageJ.

### Comparison of LARG-S1288 A and LARG-S1288A-CAAX localization and RhoA activation

A LARG-S1288 A plasmid with an added HRAS CAAX motif (CMSCKCVLS) on the C-terminal region was synthesized by Thermofisher Scientific (Thermofisher, Waltham, MA). For TIRF analysis, U87 MG cells were transfected with either LARG-S1288A or LARG-S1288A-CAAX on an 8-well chamber slide, before being serum-starved in 0.1% FBS DMEM overnight. Cells were stimulated with 100 ng/ml EGF for 0, 10 or 120 min, then fixed and stained for Myc-tag expression prior to TIRF imaging. PLA was used to compare LARG-RhoA interactions. For this, U87 MG cells were transfected with either LARG-S1288A or LARG-S1288A-CAAX on an 8-well chamber slide. Cells were serum-starved overnight in 0.1% FBS DMEM prior to 10 min treatment of 100 ng/ml EGF, at which time the cells were fixed, and the PLA analysis was performed as previously described. RhoA-GTP activation was assessed *via* ICF. U87 MG cells were seeded on 8-well chamber slides, transfected with LARG-S1288 A or LARG-S1288A-CAAX, serum-starved overnight and treated with 100 ng/ml EGF for 120 min. Cells were then fixed and stained for myc-tag expression and RhoA-GTP, before imaging and analysis was performed as previously described.

### Statistical analysis

The data were analyzed using GraphPad Prism eight and are presented as means ± SEM. Student’s *t* test (two-tailed) was used for comparisons between two conditions, and *p*-values less than 0.05 were considered statistically significant.

## Data availability

All data described are contained within this article or the supporting information.

## Supporting information

This article contains supporting information.

## Conflict of interests

The authors declare that they have no conflicts of interest with the contents of this article.

## Supporting information

Table S1

Figure S1 caption
