# Supplementary material for: Phosphorylation at S1288 of leukemia associated RhoGEF (LARG/ARHGEF12) induces plasma membrane localization and promotes binding and activation of RhoA
Source: J Biol Chem. 2025 Dec 1;302(1):110996. doi: 10.1016/j.jbc.2025.110996 (PMC12799961; doi:10.1016/j.jbc.2025.110996)
Supplement: Table S1 [file mmc1.docx]

Supp. Table1. Sex, age, and genomic data of GBM patients

| Sample # | Gender | Patient Age at Collection | Patient genomic data | | | | | | |
| --- | --- | --- | --- | --- | --- | --- | --- | --- | --- |
|  |  |  | CDKN2A | EGFR | IDH1 | PDGFRA | PIK3CA | RB1 | TP53 |
| 1 | Male | 66 | WT | c.2319_2320insCAC | WT | WT | WT | WT | V157F, P72R |
| 2 | Female | 67 | WT | WT | WT | WT | WT | WT | WT |
| 3 | Female | 59 | W110* | A289V | WT | WT | WT | WT | WT |
| 4 | Male | 70 | WT | A289T | WT | WT | I391M | WT | WT |
| 5 | Female | 69 | WT | WT | WT | WT | G127R | WT | WT |
| 6 | Female | 32 | WT | WT | WT | WT | WT | WT | R337C |
| 7 | Female | 43 | WT | WT | WT | WT | I391M | WT | R280I |

- * refers to early termination of the gene
