# Supplementary material for: Phosphorylation at S1288 of leukemia associated RhoGEF (LARG/ARHGEF12) induces plasma membrane localization and promotes binding and activation of RhoA
Source: J Biol Chem. 2025 Dec 1;302(1):110996. doi: 10.1016/j.jbc.2025.110996 (PMC12799961; doi:10.1016/j.jbc.2025.110996)
Supplement: Figure S1 caption [file mmc2.docx]

**Supplemental Figure 1. Mutant LARG-S1288A retains CDK phosphorylation.** To assess whether the mutated LARG-S1288A retained the capacity to be phosphorylated at alternative sites apart from S1288, U87MG cells were transfected with Myc-LARG-WT, Myc-LARG-S1288A, or Myc-EV, and cells were treated with 1µM nocodazole for 16 hours. Immunoblotting was done to assess the phosphorylation of CDK substrates, which demonstrated a similar increase in CDK-mediated phosphorylation of the myc-tagged LARG following nocodazole treatment in both LARG-WT and LARG-S1288A.
